# Peutz-Jeghers Syndrome Presenting With Anemia: A Case Report

**DOI:** 10.7759/cureus.26481

**Published:** 2022-07-01

**Authors:** Sidra Shakil, Zackery Aldaher, Louis DiValentin

**Affiliations:** 1 Internal Medicine, Alabama College of Osteopathic Medicine, Dothan, USA; 2 Internal Medicine, Regional Medical Center Anniston, Anniston, USA

**Keywords:** genetics, anemia, gastrointestinal bleeding, gastric polyps, peutz-jeghers syndrome

## Abstract

Peutz-Jeghers syndrome (PJS) is a rare autosomal dominant condition characterized by hamartomatous polyps, primarily in the gastrointestinal tract and mucocutaneous pigmented macules. PJS patients are at an increased lifetime risk of malignancies and complications, such as gastrointestinal bleeding from polyposis. Routine screening is critical in patients diagnosed with PJS in order to avoid complications. We report a case of a 30-year-old female with PJS who had no family history presenting acutely due to gastrointestinal bleeding and poor surveillance of her condition.

## Introduction

Peutz-Jeghers syndrome (PJS) is a rare condition characterized by mucocutaneous pigmentation and hamartomatous polyps, most commonly in the small intestine [1]. The mucocutaneous pigmentation occurs in 95% of affected individuals [2]. Although PJS hamartomatous polyps most commonly occur in the small intestine (in the order of prevalence: jejunum, ileum, and duodenum), they can also occur in the stomach, large bowel, and, in rare cases, extraintestinal sites including bronchus, renal pelvis, and urinary bladder. PJS is diagnosed based on clinical findings of having two of the three following criteria: family history, multiple pigmented macules on the mucous membranes and skin, and hamartomatous intestinal polyps [3].

Although it is an autosomal dominant inherited disorder, PJS demonstrates a prevalence of one in 100,000 individuals [3,4]. The syndrome appears equally in both genders and among all racial groups. The average age of diagnosis is 23 years in men and 26 years in women [2]. Mutations in the serine-threonine kinase 11 (STK11) tumor suppressor gene have been linked to PJS and are detected in 50-80% of families with PJS [3,4]. The rate of spontaneous mutation of this disorder is unknown and PJS resulting from de novo mutations has been reported [2,3].

Complications of PJS include intussusception or obstruction of the gastrointestinal lumen by the polyps. The polyps may also cause chronic bleeding leading to anemia [3]. In addition, the relative risk of dying from gastrointestinal cancer is 13 times greater in PJS patients compared to the general population, and these patients have a nine times greater risk for any other malignancy as well [5].

This report describes the case of a 30-year-old Caucasian female with a history of PJS with poor surveillance. She presented to her primary physician with complaints of fatigue and her labs revealed severe anemia. The patient had no family history of PJS, and upper endoscopy revealed a predominance of polyposis in the proximal body of the stomach. This case further highlights the importance of adhering to current surveillance recommendations and prevention strategies in patients with PJS in order to avoid complications.

## Case presentation

A 30-year-old obese Caucasian female with a history of PJS presented to the emergency department (ED) with the chief complaint of fatigue for three months. She had also felt dizzy and lightheaded recently and was experiencing shortness of breath with activity. She also complained of diffuse relapsing-remitting lower abdominal pain over the same period of time, which may have been related to secondary intussusception to intestinal polyps. The patient had initially gone to her obstetrician-gynecologist who had sent her to the ED, after reviewing her lab results, for further workup of her anemia. She denied any nausea, vomiting, constipation, diarrhea, melena, dysphagia, or recent weight loss.

**Figure 1:**
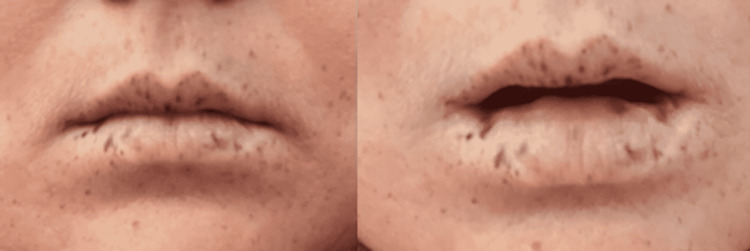
Multiple hyperpigmented spots located on lips

Upon further history-taking, the patient admitted that she had seen some blood in her stools, which was typical for her. She was admitted for further evaluation and transfusion of red blood cells. Upon physical examination, there was mild tenderness to palpation of the abdomen. Abdominal guarding, rebound tenderness, or any palpable masses were absent. The patient did have mucocutaneous hyperpigmentation on her upper and lower lips (Figure 1). No other skin lesions or pertinent physical examination findings were noted.

The patient had been clinically diagnosed with PJS at five years of age by her dentist, who had recommended she got the mucocutaneous lesions examined by a physician. Around the same time, she had been hospitalized for three months due to uncontrollable vomiting, which had led her to be evaluated for PJS. There had been no prior family history of PJS, cancers, or other gastrointestinal conditions. It was not until her first pregnancy at 24 years old that she had undergone a genetic screening for the STK11 mutation, which had turned out to be positive. However, her son’s genetic testing results had not shown the mutation.

Upon presentation, the patient was afebrile with a blood pressure of 147/86 mmHg and a pulse of 109 bpm. Red blood cells, hemoglobin, hematocrit, and mean corpuscular volume were low (Table 1). Total iron-binding capacity was high and iron saturation percentage was low. Fecal occult blood testing was positive (Table 2). Hemoglobin and hematocrit were ordered every 12 hours. The patient received two units of packed red blood cells as needed as well as an iron infusion.

**Table 1:** CBC with differentials at the time of admission CBC: complete blood count; WBC: white blood cells; RBC: red blood cells; MCV: mean corpuscular volume

Variables	Patient values	Normal range
WBC	8.3	4.5-10.4 x 10^3^/uL
RBC	3.03	3.70-5.30 x 10^6^/uL
Hemoglobin	6.6	11.0-16.0 gm/dL
Hematocrit	21.6%	35.0-47.0%
MCV	71.4	81.0-97.0 fL

**Table 2:** Further laboratory studies at admission TIBC: total iron-binding capacity; FOBT: fecal occult blood test

Variables	Patient values	Normal range
Iron	60	50-170 mcg/dL
TIBC	496	250-450 mcg/dL
Transferrin	333	204-360 mg/dL
Iron saturation percentage	12%	20-55%
FOBT	Positive	Negative

**Figure 2:**
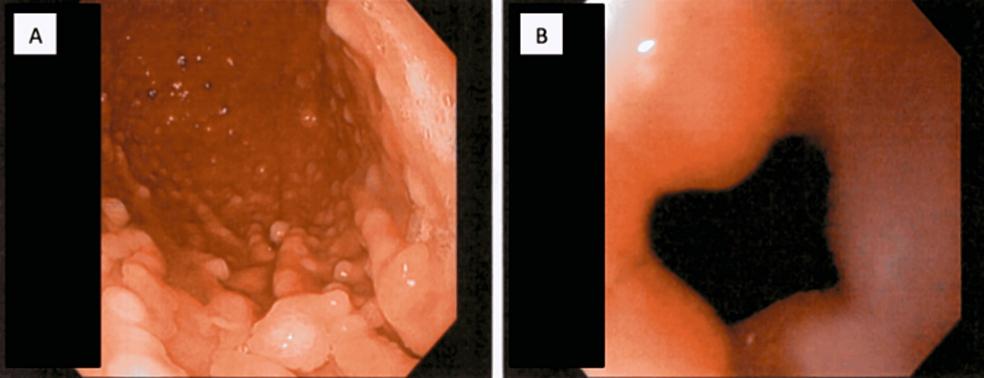
EGD showing multiple gastric polyps (A) and antral inflammation (B) EGD: esophagogastroduodenoscopy

Gastroenterology was consulted. An esophagogastroduodenoscopy (EGD) was subsequently performed, which revealed hundreds of polyps in the proximal body of the stomach, with the largest measuring 3 mm (Figure 2A). Antral inflammation was also noted (Figure 2B). The duodenal bulb and second portion of the duodenum revealed no abnormalities. Antral and gastric biopsies were taken for CLOtest, which was negative for *Helicobacter pylori.*

During the course of her four-day hospital stay, the patient’s hemoglobin and hematocrit stabilized to 8.2 gm/dL and 26.6%, respectively. She was started on anti-reflux precautions, proton pump inhibitor therapy, and iron therapy. She refused colonoscopy or further work up during her hospital stay and preferred to follow up with her gastroenterologist. She was discharged home and advised to follow up with her gastroenterologist.

## Discussion

Among the various malignancies complicated by PJS, colorectal is the most common one, with a lifetime risk of 39%, followed by breast cancers in females, with a lifetime risk of 32-54% [4]. While de novo mutations can occur, the STK11 gene localized to chromosome 19p34p36 is responsible for the vast majority of cases. This serine-threonine kinase is involved in growth control regulation [5]. The increased risk of malignancy occurs due to the hamartomatous polyps of PJS undergoing malignant change via the hamartoma-adenoma-carcinoma sequence [6].

Hamartomatous polyps are detected in 88% of patients with PJS, most commonly occurring in the small intestine (64%), colon (64%), stomach (49%), and rectum (32%). Polyps range in size from 0.1-5 cm in diameter and are typically distributed in 1-20 per segment affected. Mucocutaneous pigmentation in patients with PJS is most commonly located on the vermillion border of the lips (94% of patients) followed by buccal mucosa (64%), hands (74%), and feet (62%) [2].

PJS is most commonly diagnosed in the second decade of life, due to complications of polyposis, which include abdominal pain, bowel obstruction, intussusception, or occult gastrointestinal bleeding [4]. Intussusception of the small intestine, occurring in 47% of patients with PJS, is the most common presenting symptom leading to an initial diagnosis [2].

This case is unique for a variety of reasons. It is important to note that the patient was diagnosed as a child due to her mucocutaneous pigmentation occurring on the vermillion border of her lips and buccal mucosa. In addition, she denied any family history of PJS, and subsequent genetic testing after diagnosis revealed the presence of the mutated STK11 gene. Of note, her son lacks the presence of the gene. The patient’s polyposis was predominantly in her proximal stomach in which hundreds of polyps were revealed with the largest measuring ~3 mm. Additional workup for our patient is warranted to determine the presence of polyps elsewhere in the gastrointestinal tract. There has been a previous case reported of a patient diagnosed with an isolated PJS-type gastric polyp in the absence of any relevant family history. That patient also lacked typical mucocutaneous pigmentation [7]. In addition, isolated gastric polyposis in PJS is very rare with only 13 cases described in the literature so far [8].

Perhaps the most significant aspect of this case was the lack of surveillance and tertiary prevention strategies to avoid complications related to the patient’s condition. She had presented acutely to her obstetrician-gynecologist’s clinic for an annual follow-up with severe anemia, melanotic stools, and abdominal pain. From there, she had been sent to the ED. During her hospital stay, the patient preferred to be discharged to follow up with her gastroenterologist for further work-up, including a colonoscopy.

A meta-analysis of 210 individuals with PJS revealed a relative risk for all cancers of 15.2 and a lifetime risk of any cancer of 93% [2]. Specifically, patients with PJS have also been shown to have an increased risk of gastrointestinal malignancies [9]. It is recommended that individuals diagnosed with PJS undergo routine upper and lower endoscopy every two years [5]. In addition, polyp removal is the mainstay of therapy to prevent further complications [4]. While the patient confirmed she follows up with her gastroenterologist annually, counseling was never initiated with regard to polyp resection to avoid potential complications.

Other periodic surveillance includes routine screening for breast and pancreatic cancers. The patient revealed that she had undergone genetic testing while pregnant with her son, which confirmed the presence of the mutated gene. However, it is recommended that females with PJS planning to conceive should undergo genetic testing and counseling prior to becoming pregnant [3].

This case emphasizes the importance of patient education, routine surveillance, and tertiary prevention strategies to avoid complications in individuals diagnosed with PJS. Further studies may be warranted to determine the frequency of sporadic mutations and the proportion of patients with predominant gastric polyposis in PJS.

## Conclusions

PJS in a patient without a family history and a predominance of gastric polyposis has not been previously reported in the literature. Further research is indicated to determine the prevalence of sporadic mutations among patients diagnosed with PJS. This case also emphasizes the importance of routine screening to prevent complications, as in this case, in individuals with PJS.
